# Effective Practical Solutions for De-Icing of Automotive Component

**DOI:** 10.3390/nano12172979

**Published:** 2022-08-28

**Authors:** Andrea Tinti, Gloria Anna Carallo, Antonio Greco, María Dolores Romero-Sánchez, Luigi Vertuccio, Liberata Guadagno

**Affiliations:** 1 Consorzio CETMA, Advanced Materials & Processes Consulting Department, S.S. 7 km 706+030, 72100 Brindisi, Italy; 2 Department of Innovation Engineering, University of Salento, Via Monteroni, 73100 Lecce, Italy; 3 Applynano Solutions S.L., Parque Científico de Alicante, Naves de Apoyo 3, 03005 San Vicente, Spain; 4 Department of Engineering, University of Campania “Luigi Vanvitelli”, Via Roma 29, 81031 Aversa, Italy; 5 Department of Industrial Engineering, University of Salerno, Via Giovanni Paolo II, 84084 Fisciano, Italy

**Keywords:** acrylonitrile butadiene styrene, thermoplastic polyurethane, thermoplastic vulcanizates, multi-walled carbon nanotube, Joule heating de-icing

## Abstract

Carbon Nanotube (CNTs)-based masterbatches have been mixed with thermoplastic polymers currently used to manufacture automotive components. These mixtures have been tailored to integrate an effective heating function in the materials. The manufacturing method for composite compounding and processing conditions significantly affects the electrical resistivity of the developed materials. The resistivity of the material can be controlled within tight tolerances sufficient to meet automotive requirements. The optimal compounding parameters of the melt process technologies were defined to obtain uniform filler dispersion and distribution. Heating and de-icing tests were performed on sheet specimens with optimized CNT content and electrical conductivity suitable for effective electro-thermal behaviour with low input voltages (≤24 V DC), making them safe for users. Finally, a simplified analytical model of the Joule effect arising from an energy balance of the system under study (heat equation) was developed and validated by comparison with experimental data for use in future development for the purpose of the preliminary design of components in the automotive sector.

## 1. Introduction

In the last several years, great effort has been devoted by researchers in developing smart bulk materials for applications in the automotive sector incorporating responsiveness properties, with the aim of increasing consumer safety, component lifespan, and performance while reducing maintenance and manufacturing costs and environmental pollution. In this scenario, the development of innovative smart heating solutions oriented to increasing safety and comfort in the cold winters of North Europe can provide solutions for key vehicle critical components such as windscreen wipers, door locks, battery filling systems, external push buttons, etc., which manifest reduced performance in environments below 0 °C or thereabouts. An issue to be addressed in these environmental conditions is the implementation of de/anti-icing function in vulnerable components to avoid ice formation and related performance loss. Promising strategies have been proposed in the literature to activate de/anti-icing functions in thermo-setting resins and carbon fiber-reinforced composites (CFRCs) [1,2,3]. When the temperature and the humidity reach a certain value, the component starts to be heated by the external stimulus of an applied voltage [4]. In fact, heating and de-icing functionalities are based on the Joule effect, the process by which the passage of an electric current through a conductor produces heat [5]. Under direct current (DC) conditions, the most fundamental formula for Joule heating is the Joule’s first law (P = I·V), where P is the power (energy per unit time) converted from electrical energy to thermal energy, I is the current flowing through the resistor or other element, and V is the voltage drop across the element. Assuming that the element behaves as a perfect resistor and that the power is completely converted into heat, the formula can be re-written by substituting Ohm’s law, V = I·R, into the generalized power equation, P = IV = I^2^R = V^2^/R, where R is the resistance [6]. Traditionally, for this kind of application, e.g., in aeronautics, electric ice protection systems require the employment of metallic heating elements [7]. Recently proposed de-icing strategies based on innovative solutions have exploit the functional properties of conductive nanoparticles to reduce the weight of bulk materials in the field of civil engineering and aeronautics, in the latter case leading to a reduction in fuel consumption and pollutants [1,3]. The employed nanoparticles, namely, carbon nanotubes and graphene-based nanoparticles, confer other desirable applicative functionalities to the host polymeric matrices: (i) they are capable of increasing the durability of polymeric materials, which are vulnerable to sunlight [8,9,10,11]; (ii) they are able to enhance mechanical, electromagnetic, and dielectric properties [12,13,14,15,16,17,18]; (iii) they are well suited to revolutionizing manufacturing processes to save considerable amounts of energy [19]; and (iv) they are able to increase the adhesion properties of polymers by using carbon fabric as a filler in structural components [20,21,22].

Nanotechnology-based strategies can be advantageously applied in the automotive sector. In this field, despite several indoor and outdoor components of cars and other road vehicles being subject to icing in cold climates, resulting in comfort and safety concerns, often no or inefficient solutions are used to prevent frost problems and related malfunctions. Examples of critical automotive components wherein de-icing functionality could be incorporated with clear advantages include the door gaskets/tape for the whole car door perimeter, in order to avoid sticking in winter; the windshield cowl and other external parts such as mirror covers, to prevent snow accumulation and enhance visibility and safety; and tubes and pipeline components for water circuit systems, to guarantee regular liquid flow and functioning. In the absence of a proper ice protection system, in northern countries the most common solution is to use an electric heater inside the car. In such cases the full cab area is heated, not specific areas, leading to inefficient systems and high energy consumption. Alternatively, manual operations for snow and ice removal by hand or by means of a portable air flow heater are needed. Keeping the engine on or sheltering the car inside a garage at night are common strategies to work around these problems.

In the present work, three thermoplastic polymers commonly used in the automotive sector have been studied: thermoplastic vulcanizate polymer (TPV), acrylonitrile butadiene styrene (ABS), and thermoplastic polyurethane (TPU). De-icing function has been conferred on these polymers by incorporating electrically conductive nanomaterials into the base matrix. Multi-wall carbon nanotubes (MWCNTs) were used in the form of compatible thermoplastic masterbatches. We have obtained very promising results, which permit for a glimpse of many easy and interesting practical applications.

## 2. Materials and Methods

### 2.1. Materials

CNT-based masterbatches suitable for diluting thermoplastic polymers widely used in the automotive sector were provided by Arkema (Colombes, France). The chosen masterbatches guarantee a proper dispersion of CNTs in the polymer composite, providing such relevant benefits as good dispersion, easy handling, and remarkable electrostatic dissipation (ESD) properties. They belong to the class of Graphistrength^®^ and are based on MWCNTs obtained by catalytic chemical vapor deposition using corn-origin bioethanol as a carbon source in an industrial fluidized bed system yielding 400 T/yr. They are characterized by an outer mean diameter ranging between 10 nm and 15 nm, a length in the range from 0.1 μm to 10 μm, a carbon content higher than 90 wt%, and an Aspect Ratio (AR) ranging from 10 to 1000.

Three thermoplastic polymers commonly used in the automotive sector were used: thermoplastic vulcanizate polymer (TPV), acrylonitrile butadiene styrene (ABS), and thermoplastic polyurethane (TPU). Table 1 lists the pure thermoplastic polymers and CNT-based masterbatches along with information on the composition adopted for developing electro-thermal plastic components for automotive applications.

Graphistrength^®^ C M14-25 masterbatch is a commercially available product composed of 25 wt% of MWCNTs dispersed in a polypropylene matrix. It contains no processing aids or other additives.

Graphistrength^®^ C ABS1-17 masterbatch is a commercially available product composed of 17 wt% of MWCNTs dispersed in a matrix of ABS.

Graphistrength^®^ C TPU1-20 masterbatch is a commercially available product composed of 20 wt% of MWCNTs dispersed in polyester-based thermoplastic polyurethane matrix without any other additives. It is particularly suitable for mixing with polyurethane materials.

Specific thermoplastic matrices suitable for potential automotive applications were selected for each CNT-based masterbatch mentioned above (see Table 1).

CNT-based masterbatch M14-25 was used to confer electro-thermal properties on a TPV thermoplastic matrix. TPV polymer is an emerging material suitable for the gasket production market as an alternative to thermoset rubbers alone, and is based on ethylene propylene diene monomer (EPDM). TPV polymer is a thermoplastic vulcanizate which belongs to a specific class of thermoplastic elastomers; it is made of a matrix of polypropylene (PP) with finely-dispersed pre-cured EPDM particles. The dispersed phase causes the material to behave similarly to rubber, while the continuous phase allows the material to be processed as a thermoplastic polymer. A proper commercial variety of neat unfilled TPV polymer specially recommended for automotive applications was selected. It combines the characteristics of vulcanized rubber with the processing properties of thermoplastics. The commercial name is Santoprene™ 121-58W175 from Exxon Mobil. The material has a Shore A hardness of 62, which is in the useful range for, e.g., a gasket component. Moreover, the brittleness temperature is −60 °C; hence, the product maintains its softness and flexibility properties at freezing temperatures. The neat material is an electrical insulator with a dielectric strength of 25 kV/mm.

CNT-based masterbatch C ABS1-17 has been used to confer electro-thermal property on ABS matrix. This polymeric matrix is commonly used in automotive interiors and exteriors, e.g., windshield cowls and mirror covers. As the ABS host matrix, UV-resistant grade Starex^®^ SV-0167 from Cheil Industries was selected.

CNT-based masterbatch C TPU1-20 has been used to confer electro-thermal properties on a TPU matrix belonging to the family of thermoplastic elastomers. TPUs are very often used together with plasticized polyvinyl chloride (PVC) in the manufacturing of flexible tubes. The selected neat TPU grade was Elastollan^®^ 1180 A 10 from BASF, which is a polyether polyurethane with outstanding low temperature flexibility and a Shore A hardness of 80, which is in the useful range for, e.g., pipeline components.

### 2.2. Production of Nano-Composites

Nanocomposites were produced with a single-screw (SS) extruder (see Figure S1 of the Supplementary Materials). For each kind of polymer (TPV, ABS, and TPU) mixed with the masterbatches, a number of different mixtures were produced with different compositions and optimized setting parameters. Detailed information on the production of the nanocomposites is provided in the Supplementary Materials, “Production of the nanocomposites and processing parameters”.

For the TPV sample mixed with C M14-25 masterbatch, compositions containing different percentages of MWCNTs were prepared. These were named with the acronyms TPV(x), where x is the percentage of MWCNTs.

For the ABS sample mixed with C ABS1-17 masterbatch, compositions containing 0, 5, and 12 wt/wt percentage of MWCNTs were prepared. These were named with the acronyms ABS(0), ABS(5), and ABS(12), respectively.

Similarly, for the TPU sample mixed with C TPU1-20 masterbatch, compositions containing a percentage of MWCNTs from 0 to 12 wt/wt were prepared. These were named with the acronyms TPU(x), where x is the percentage of MWCNTs.

### 2.3. Methods

In the present work, nano-composite characterization was carried out in order to evaluate the uniformity of filler distribution. This was done by both direct methods such as transmission electron microscopy (TEM) and indirect ones such as mechanical, rheological, and electrical measurements.

The dispersion of the filler in the polymeric matrix was investigated by TEM using a JEOL model JEM-1400 Plus microscope equipped with an image acquisition camera model GATAN (ORIUS) and a resolution of 0.38 nm between dots and 0.2 nm between lines. Thin films of each sample were prepared for the TEM microscope using an ultramicrotome (RMC, model MT-XL) with dimensions 1 mm × 1 mm and 80–100 nm thickness. Samples were deposited on a copper grid by means of lacey carbon films for TEM analysis.

Mechanical analysis was carried out by means of tensile testing to evaluate material embrittlement upon high loading with nano-fillers, which can result in reduced material performance as concerns strain at break. To this end, a Lloyd LR 50K was used; 70 mm × 10 mm × 2 mm samples were subjected to a uniform rate of deformation using a cross-head speed of 50 mm/min.

Rheological tests were performed to evaluate the increase in melt viscosity upon addition of CNTs, which can affect material processing during both the compounding step and subsequent forming step for the manufacturing of, e.g., automotive items. An ARES Rheometrics system equipped with 25 mm parallel plates geometry was used. Steady state tests were performed with a shear rate between 0.05 and 1 s^−1^.

Electrical conductivity was measured in order to determine the percolation curve and the condition of the conductivity plateau. In fact, a filler concentration beyond the Electrical Percolation Threshold (EPT) is suggested to maximize the Joule effect [3].

**Figure 1:**
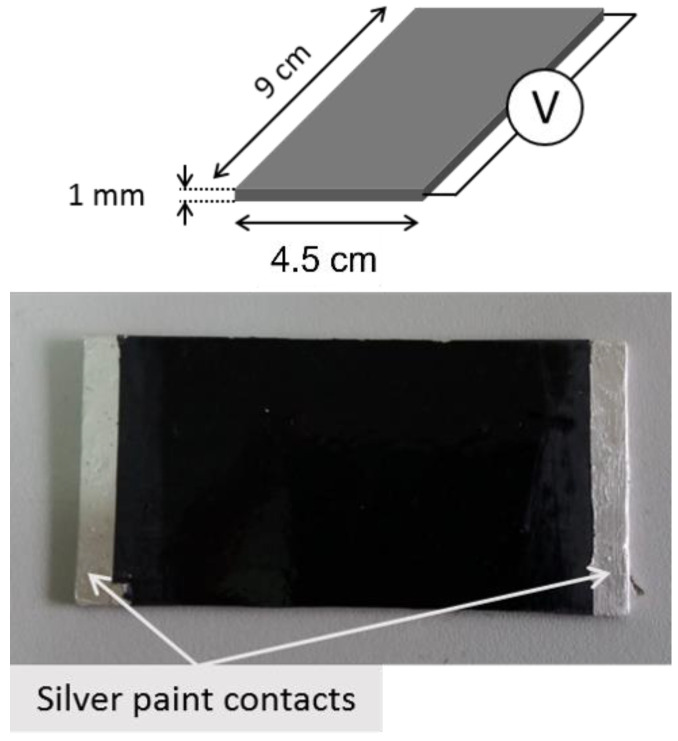
Specimen geometry for evaluating electrical and responsivity measurements.

Electrical measurements were performed on rectangular specimens with about 1 mm thickness and 45 mm × 90 mm sides. In order to reduce possible effects due to surface roughness and to ensure ohmic contact with the measuring electrodes, the samples were coated using a silver paint with a thickness of about 50 μm and characterized by a surface resistivity of 0.001 Ω cm (Figure 1). The measurement apparatus for DC characterization of samples above the percolation threshold consisted of a multimeter Keithley 6517A with a voltage generator function (maximum ±1000 V), voltmeter (maximum ±200 V), and ammeter HP34401A (minimum current 0.1 µA). The I-V characteristic between 0 and +25 V (+15 V for the most conductive specimens) was collected, and the specimen resistance was calculated as the slope of the linear curve. Below the percolation threshold the system was composed exclusively by a multimeter Keithley 6517A working as voltage generator and pico-ammeter (minimum current 0.1 fA). Here, a single measurement at a constant voltage (100 V) was made and the specimen resistance was calculated from Ohm’s law.

After complete development and characterization of the materials of interest (TPV, ABS, and TPU), we continued by analyzing the responsiveness measurements. Heating/de-icing tests were performed on sheet specimens resulting from the cast extrusion compounding process. For each material, we considered the most promising %CNT in terms of the resulting electrical conductivity in order to effectively heat the material (reasonable temperature and heating time) considering the low input voltages typically available onboard a car (the typical car battery is 12 V DC, although in work vehicles and sports cars 24 or 48 V DC batteries are commonly available).

**Figure 2:**
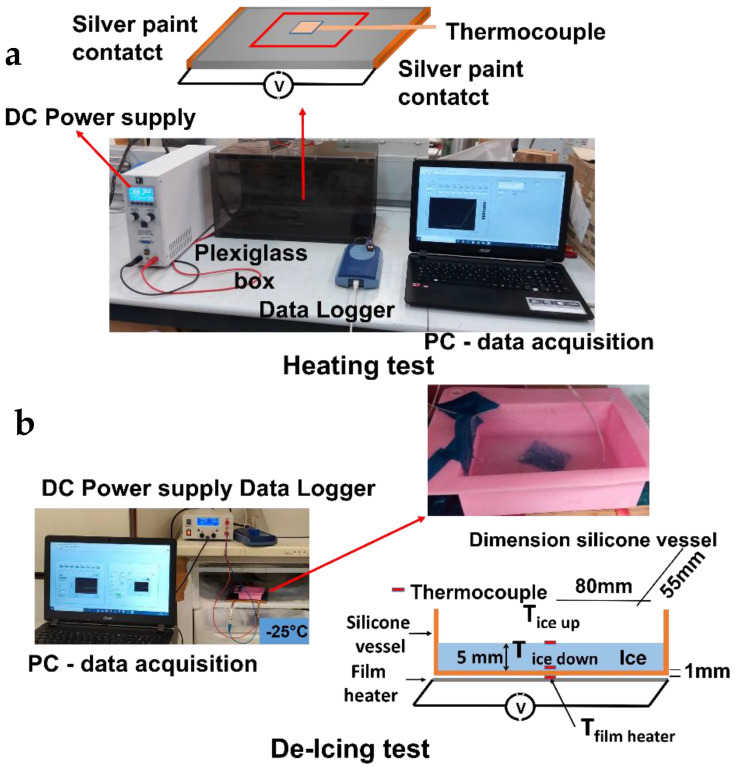
Heating measurement layout: (**a**) schematic representation of the heating tests and (**b**) configuration of the de-icing tests.

Heating tests were conducted at room temperature (25 °C) by applying a constant voltage of 12 or 24 V DC and continuously recording the material temperature vs. time profile. A data acquisition board (Thermocouple Data Logger, supplied by Pico Technology, St Neots, UK) was used to acquire the thermocouple measurements. In order to record the local temperature evolution, dedicated LabView software was developed. The temperatures were measured using thin wire thermocouples (Type K Omega Engineering Ltd., Manchester, UK) with negligible thermal inertia, which were positioned at the centre of the sample. The laboratory power supply was an EA-PSI 8360-10T (Elektro-Automatik, 0–360 V, 0–10 A, 1000 W max), which was connected to the sample as described in Figure 2.

Based on the heating results, de-icing tests were performed at selected voltage values for each kind of material under study. In this case, the external temperature was about −25 °C. A 5 mm thick slab of ice was frozen on the specimen surface to evaluate de-icing effectiveness (Figure 2). Further details of the experimental layout are reported elsewhere [2,3].

## 3. Results

### 3.1. TEM Micrographs

**Figure 3:**
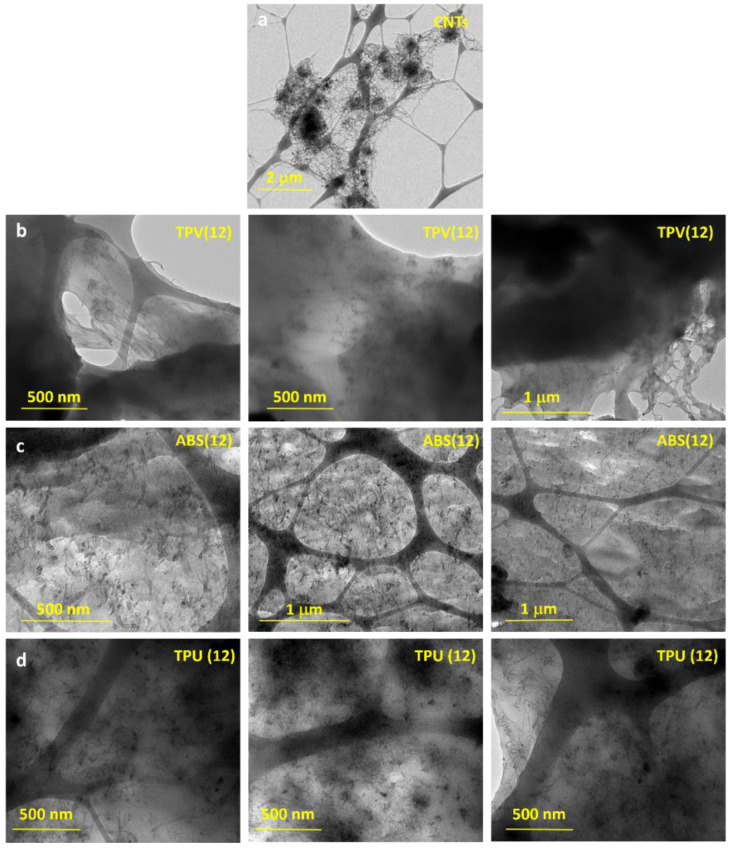
TEM micrographs of samples: (**a**) CNTs; (**b**) TPV(12) nanocomposite; (**c**) ABS(12) nanocomposite; (**d**) TPU(12) nanocomposite.

TEM micrographs were captured for the CNT samples alone and for the nanocomposites with the highest CNT content (apart from the neat masterbatch) for each material family. These are shown in Figure 3.

Figure 3 shows that the entanglement of CNTs when incorporated in the polymeric matrices is lower compared to the entanglement observed for the CNTs alone (Figure 3a). This is clear evidence that the mixing process performed during nano-composite preparation allows for better separation and dispersion of the CNTs in the hosting matrices, while the morphology of the CNTs seems to be unmodified. Hence, good electrical conductivity of the filled polymeric matrices can be expected.

### 3.2. Tensile Tests

Tensile tests for each composition of the developed nanocomposites were performed.

**Figure 4:**
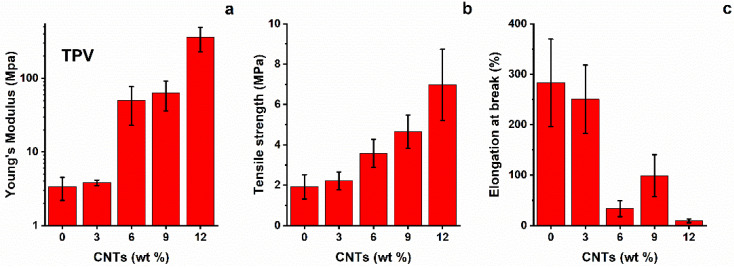
(**a**) Young’s modulus, (**b**) tensile strength, and (**c**) strain at break of TPV nanocomposites.

The average results of the tensile tests for the TPV(x) system are shown in Figure 4. It is clear that the presence of nanotubes from 6%wt to 12%wt strongly affects the mechanical properties. In fact, the Young’s modulus changes from that of a rubbery material, ≈3–5 MPa for TPV(0) and TPV(3), to that of a semi-rigid material, ≈100 MPa, starting from TPV(6) up to TPV(12). This trend is confirmed in the evaluation of the other tensile properties, i.e., tensile strength and strain at break. A progressive increase in tensile strength with increasing content of nanotubes is observed. Indeed, it is possible to observe a drastic reduction in strain at break for nano-composites with higher CNT percentages, with values that decrease of about six times with respect to that of TPV(0) and TPV(3). This is indicative of an increase in brittle behaviour shown by nanocomposites with increasing content of nanotubes. This is an expected behaviour due to the reduced chain mobility determined by the nanotube network [22].

**Figure 5:**
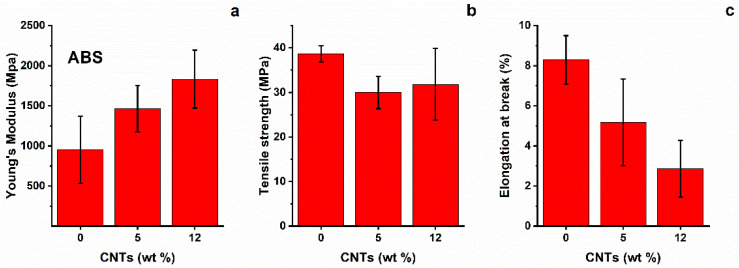
(**a**) Young’s modulus, (**b**) tensile strength, and (**c**) strain at break of ABS nano-composites.

ABS outcomes are presented in Figure 5. The general trend of tensile modulus is in agreement with the data from the literature [23], showing a continuous increase with CNT content up to 12%wt. On the other hand, tensile strength does not increase with CNT content. A possible explanation for this behaviour (the literature suggests an increase in strength up to 10%wt nanotubes, with the maximum reinforcing effect between 1 and 7%wt) is that it can be attributed to clusters of nanotubes which produce a filler–filler interaction; this leads to a reduction in interfacial adhesion, with resulting internal shear delamination and lower tensile strength [24]. However, the TEM micrographs show a quite satisfactory distribution of nanotubes on the polymer bulk, at least in the selected areas of point analysis. Finally, strain at break shows a progressive decrease with increasing CNT content, highlighting the brittle behaviour of nanocomposites. The strain results are in good agreement with the data reported in the literature [24].

**Figure 6:**
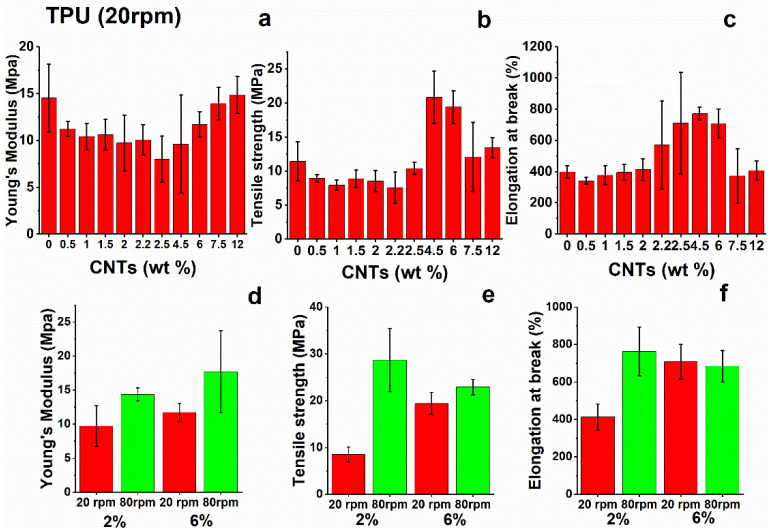
(**a**) Young’s modulus, (**b**) tensile strength, and (**c**) strain at break of TPU nanocomposites; (**d**–**f**) tensile parameters at different values of speed rotation (rpm) during the mixing step.

The tensile test results for TPU nanocomposites are shown in Figure 6. Mechanical analysis shows that the addition of nano-filler to the neat matrix does not provide a remarkable increase in the tensile modulus of the nanocomposites. Instead, intensive mixing produces slight improvements in the modulus as result of better dispersion of the filler into the TPU-based matrix. Similar considerations apply to the analysis of tensile strength and strain at break values of TPU nanocomposites. These results confirm the previous statements, in particular, the role of intensive mixing, which seems to positively influence the dispersion of nanotubes and give rise to better results with respect to the same nanocomposites produced with standard mixing conditions.

### 3.3. Rheological Measurements

The nanocomposites underwent rheological analysis for evaluation of their dynamic viscosity. The test temperatures were fixed close to the extrusion temperature (die), as reported in Tables S1–S3 of the Supplementary Materials.

**Figure 7:**
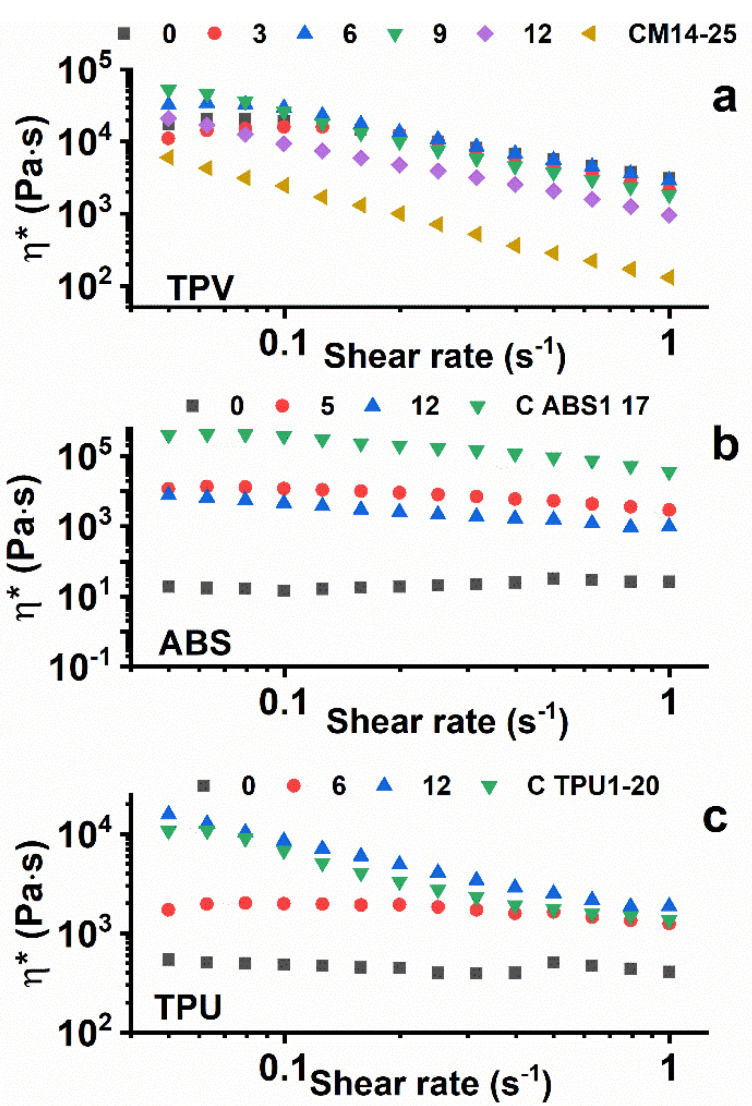
Viscosity vs. shear rate of TPV(x) (**a**), ABS(x) (**b**), and TPU(x) (**c**) at different loadings of MWCNTs.

Figure 7 shows the viscosity vs. the shear rate for the three analyzed samples, TPV(x) (Figure 7a), ABS(x) (Figure 7b), and TPU(x) (Figure 7c), at different loadings of MWCNTs.

For TPV(x) materials, the measurements were carried out at T = 200 °C. As expected, nanocomposites embedding low amounts of CNTs manifest viscosity values similar to neat TPV. The more highly charged ones, in particular the TPV(9) sample, show higher viscosity values at a near-zero shear rate. The lower viscosity of TPV(12), which seems not to follow this trend, can be explained as a consequence of increasing the content of masterbatch, the matrix of which has a lower viscosity with respect to that of neat TPV, determining a reduction in the viscosity of the final nano-composite.

Rheological analysis on ABS nanocomposites was performed at T = 230 °C. With the exception of neat ABS, which has very low viscosity (≈10^1^ Pa∙s), the other nanocomposites show higher viscosity, in the order of magnitude of 10^4^ Pa∙s, due to the addition of nanotubes. The neat MB C ABS1-17 exhibits the highest viscosity of the ABS family (>10^5^ Pa∙s).

Rheological analysis of TPU nanocomposites was performed at a temperature of 195 °C. For readiness of comprehension, only significant curves are reported in the viscosity vs. shear rate graph. It is possible to notice that the adding of nano-filler produces an increase in viscosity with respect to the neat TPU matrix, as was expected. Indeed, the nanocomposite with the maximum percentage of nanotubes added, 12%wt for TPU(12), presents a viscosity approaching that of the neat masterbatch, with an order of magnitude of 10^4^ Pa∙s.

For the neat polymers ABS(0) and TPU(0), the graphs of the complex viscosity clearly evidence Newtonian behaviour. In fact, η* manifests an almost constant value in the analysed range of shear rate. The TPV(0) sample is instead shear-dependent with respect to the ABS(0) and TPU(0) samples. For the TPV system, the masterbatch (C M14-15) is clearly below the other curves. Hence, for this last system the increase in viscosity is due to the higher viscosity of sample TPV(0) with respect to that of the masterbatch alone. Considering the behaviour of the nanocomposites belonging to the other two families, well-percolated structures are observed for a concentration of CNTs higher than 5% for the ABS(x) system and 6% for the TPU(x) system. This behaviour seems to agree well with the results of the percolated structure from the electrical point of view. Very similar results have been obtained in the literature for nano-composites based on thermosetting matrices [3,25,26].

### 3.4. Electrical Conductivity Measurements

**Figure 8:**
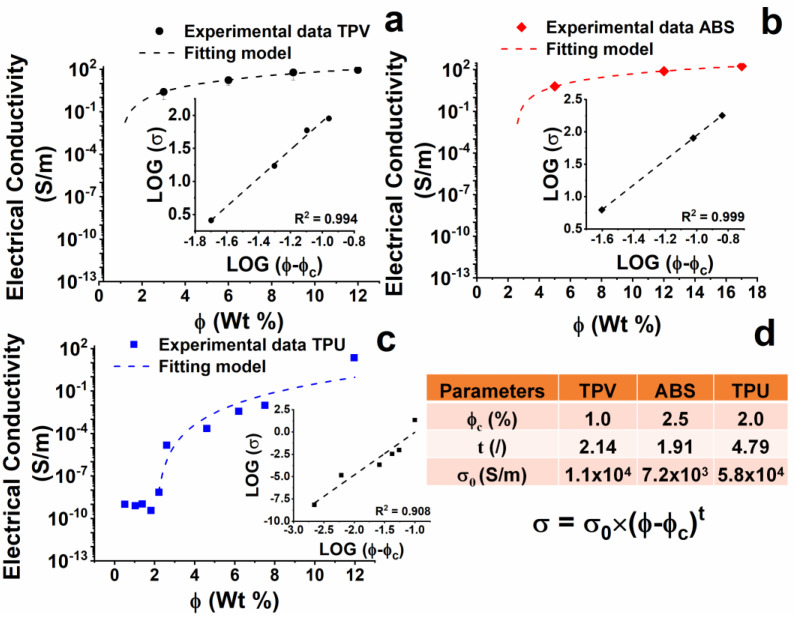
Electrical conductivity of produced nano-composites and results of percolative theory applied to the experimental dataset of the samples: (**a**) TPV(x); (**b**) ABS(x); (**c**) TPU(x); (**d**) Fitting parameters of the percolative theory.

The results on the electrical properties of the developed nano-composites are shown in Figure 8.

The TPV(x) samples (see Figure 8a) show a continuous increase in electrical conductivity with increasing the concentration of CNTs. The EPT is placed around the concentration of 1% by weight of CNTs. Among the analyzed samples, the maximum value of electrical conductivity is 8.9 × 10^1^ S/m, detected for the TPV(12) sample.

Results related to the ABS(x) sample are shown in Figure 8b. An almost percolative behaviour with a threshold around 2.5% by weight of CNTs is observed. The maximum value of electrical conductivity is 1.8 × 10^2^ S/m, detected for the ABS(17) sample, which is very similar to the electrical conductivity of the ABS (12) sample.

The results for the TPU nanocomposites are reported in Figure 8c. TPU nanocomposites do not show the typical percolative behaviour (see “Percolation theory” in the Supplementary Materials). However, an approximative value of EPT can be estimated at around 2% by weight of CNTs, which is higher than that found for the TPV(x) system.

All nanocomposites’ electrical conductivities and percolation thresholds were used as variables in Equations (S1)–(S3) (see Supplementary Materials), giving rise to the insert graphs (fitting parameters) reported in Figure 8a–c and in Table of Figure 8d, where the *σ*_0_ (S/m) value and *t* (dimensionless) exponent are reported for all the analyzed systems.

It is worth noting that for the formulated nanocomposites, increasing the percentage of MWCNTs consistently changes the chemical nature of the hosting matrix, as MWCNT are added together with the masterbatch matrix, which is chemically different from the nanocomposite matrix. It is very likely that deviations from the expected profile based on the percolation theory described in the related section of the Supplementary Materials and by other authors in literature [19,22,27] are due to this occurrence, which is not common in scientific manuscripts on this topic. This deviation is consistently observed for the TPU(x) system, for which no plateau condition is reached in the log scale [28,29,30].

### 3.5. Heating and De-Icing Tests

#### 3.5.1. Simplified Analytical Model of Joule Effect

In this paragraph, a brief introduction of the Joule effect phenomenon and theory is provided, followed by a description of the analytical model used to characterize the developed nanocomposites for automotive applications. Later, the model is applied to each material in order to deduce the maximum temperature achieved in a certain time when using the setting parameters (voltage) and properties of the specific nanocomposite.

The Joule effect is an energy dissipation phenomenon which involves electrical current-carrying conductors. It irreversibly converts electrical energy produced by the passage of electrical current in a conductor material into thermal energy [31]. The relationship between the heat generated and the current flowing into a conductor can be expressed using the formula known as Joule’s first law, reported below:

(1) $$P\;=\;I\cdot V=\frac{V^{2}}{R}$$

where I (A) is the electrical current flowing into the conductor by means of a voltage V (V) applied to its ends; their product (i.e., electrical power) is converted in P (W), thermal power, due to the heating of the material, and R (Ω) is the electrical resistance of the material towards the current flow.

The volumetric rate of Joule heating, which is useful for numerical model construction, uses the electrical conductivity *σ* (S/m), an intrinsic material property correlated to resistance R (Ω) by means of the geometrical relationship

(2) $$R=\frac{L}{\sigma \cdot A}$$

where L (m) is the distance between the voltage terminals applied and A (m^2^) is the cross-section surface perpendicular to the electrical current flow. Then, Equations (1) and (2) can be combined to obtain the power generated per unit volume, as follows:

(3) $$P_{v}=\frac{V^{2}\cdot \sigma }{L^{2}}R$$

Equation (3) is known as the volumetric rate of Joule heating (W/m^3^) or electrical power source.

The theory of the Joule effect, on which the model is based, starts from the description of the differential volume thermal energy conservation equation related to heat conduction; for the purposes of the present study, it is possible to neglect convection, because it is the fundamental transfer process in liquids, as well as radiation, because it becomes influential only at high temperatures (depending the flux by the fourth power of temperature, according to Stefan–Boltzmann radiation theory).

This statement allows us to write the heat diffusion equation in three-dimensional form as follows:

(4) $$\rho \cdot c_{p}\frac{dT}{dt}=\nabla \cdot k\nabla T+\frac{V^{2}\cdot \sigma }{L^{2}}$$

where *ρ* (kg/m^3^), *k* (W/m∙K), and *c*_*p*_ (J/kg∙K) are the density, thermal conductivity, and specific heat of the material, respectively, while *T* (K) is the temperature and *t* (s) is the time. Equation (4) states that the variation of temperature in time (the left term, called heat flux or heat power) is proportional to the 3D temperature gradient and power generating source (right terms).

**Figure 9:**
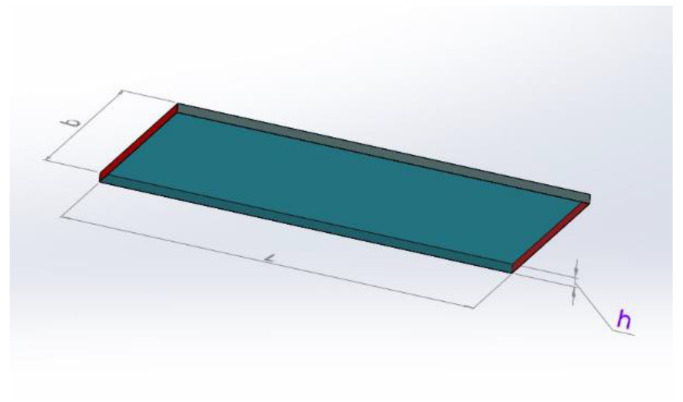
Representative volume of Joule effect model.

The analytical model is derived from the integration of Equation (4) into a volumetric domain represented by a slender plate of material with rectangular cross-section having L and b (m) as sides and h (m) as thickness, where A = b × h. A representative volume for the domain is shown in Figure 9. In the picture, the red surfaces are those on which the voltage terminals are applied, while all external surfaces are involved in convective heat exchange with the air.

The boundary conditions for Equation (4) are represented by the convective transport equation, known as Newton’s law of convection, i.e.,

(5) $$-k\cdot \nabla T=h_{c}\left(T-T_{ext}\right)$$

where −*k*∙∇*T* (W) is the thermal flux, *T* (K) is the final temperature of the material, *T*_*ext*_ (K) is the external temperature of the environment in which the domain is merged, and *h*_*c*_ (W/m^2^∙K) is the convective heat transfer coefficient of the air.

Thus, considering Newton’s law of convection as a boundary condition and neglecting the temperature gradient of Equation (4) due to almost uniform temperature on the plate volume, it is possible to rewrite Equation (4) as follows:

(6) $$Mc_{p}\frac{dT}{dt}=\frac{V^{2}\sigma A}{L}-h_{c}A_{c}\left(T-T_{ext}\right)$$

where *M* (kg) is the mass of the material (obtained by the product between density and volume) and *A*_*c*_ (m^2^) is the surface involved in convective fluxes.

By integrating Equation (6), it is possible to obtain the following closed-form solution for Equation (4):

(7) $$T=T_{ext}+\frac{V^{2}\sigma A}{h_{c}A_{c}L}\left[1-exp\left(-\frac{h_{c}A_{c}}{Mc_{p}}t\right)\right]$$

This equation defines the temperature profile along the analysed sample, and is the outcome of the analytical model employed in this work.

When validated by comparison with available experimental data, the model can be used to predict the heating behaviour of the materials of interest in any size/geometry and operating conditions, i.e., for the purpose of preliminary component design.

#### 3.5.2. Experimental Data and Comparison with the Model

**Figure 10:**
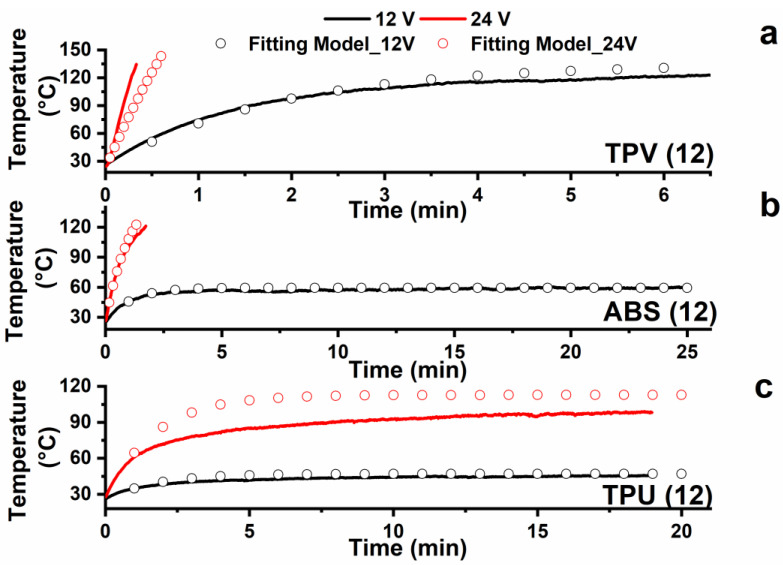
Comparison between experimental data (continuous curves) and analytical model (empty circles) of heating results detected at constant DC voltage on sheet-shaped specimens (**a**) TPV12; (**b**) ABS12; (**c**) TPU12.

Figure 10 shows the experimental curves (continuous curves) of temperature vs. time recorded during the heating test starting from room temperature (25 °C) together with the behaviour of the curves obtained applying the developed model (empty circles). The heating tests were performed considering the samples for which the highest value of DC conductivity was detected. In particular, samples TPV(12), ABS(12), and TPU(12) were tested.

Considering the experimental measurements performed at 12 V, when the input voltage is turned on, the specimen temperature rapidly increases as a consequence of Joule effect heating. A temperature plateau is generally observed within a few minutes of testing. Furthermore, the higher applied voltage of 24 V results in a higher value of the temperature plateau (see TPU system). All the analyzed systems, namely, TPV(12), ABS(12), and TPU(12), effectively heat up with an input voltage of 12–24 V DC. Furthermore, at 24 V applied voltage, the temperature values of both TPV and ABS increased so rapidly that it was necessary to promptly stop the test in order to avoid undesired polymer matrix softening phenomena.

As shown in Figure 10, the analytical model of heating for sheet specimens (Equation (7)) fits well with the experimental data and can be used for the purpose of component design. The convective heat transfer coefficient of air (*h_c_*) was found to be one of the most important fitting parameters, and its value was fixed at 10 W/(m^2^·K), which is typical of natural convection conditions. A complete list of the fitting parameters used for the three case studies is reported in Table 2.

The fitted data for TPU in Figure 10 seem to deviate in no negligible way from the experimentally recorded data. This behavior is most likely due to the model used in this work assuming that the applied power is the same during the heating test.

If specific temperature values typical of each material are exceeded, the electrical conductivity, and therefore the electrical resistance of the material, can change with the temperature. Generally, a material can behave as appropriate with a characteristic positive temperature coefficient (PTC) effect and negative temperature coefficient (NTC) effect of resistance. This behavior occurs when the material exhibits transition phenomena due to the effect of temperature, which modifies the network of conductive particles inside the matrix by modifying the electrical resistance of the sample itself. In our case, the model predicts lower heating than that obtained experimentally. This is Most likely due to the temperature reached by the sample. The system appears to be an NTC thermistor; hence, the resistance of the sample decreases during heating, causing an increase in the current flow with the same applied voltage.

**Figure 11:**
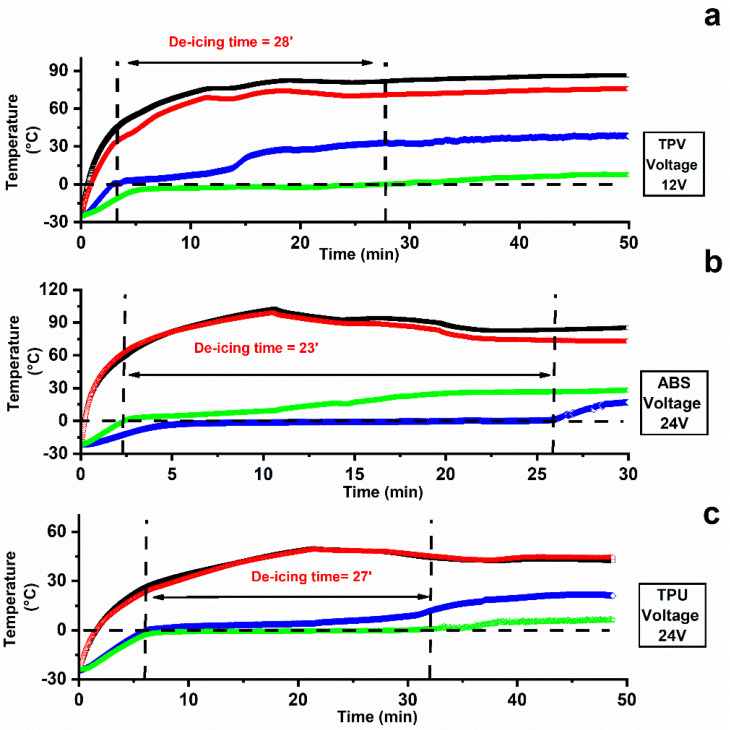
De-icing results at constant DC voltage on sheet-shaped specimens (**a**) TPV12; (**b**) ABS12; (**c**) TPU12.

Figure 11 displays the curves of temperature vs. time recorded during de-icing testing the samples TPV(12), ABS(12), and TPU(12) starting from −25 °C. The tests were performed applying different voltages. This choice was made because the tested samples reach different values of temperature in the plateau zone; see Figure 10. In particular, for the ABS and TPU samples an applied voltage of 12 V allows relatively low temperatures to be reached (45 °C for TPU and 60 °C for ABS), whereas for the TPV sample an applied voltage of 12 V allows for a higher value of temperatures (around 130 °C). Hence, for this last sample, the lower value of applied voltage was chosen.

The de-icing time, which is the time needed to melt all the ice volume (a 5 mm thick ice slab), was evaluated as the time occurrent to register a temperature exceeding 0 °C for both the first ice layer (in contact with the specimen) and the last ice layer (in contact with the surrounding air); see Figure 2b.

Figure 11 shows the surface temperatures of the active heating element (T_up_ film and T_down_ film).

De-icing tests at −25 °C were successfully completed on all the materials of interest, resulting in useful de-icing times of less than 30′ to melt the 5 mm thick ice slab at low applied voltage (12 V for the TPV(12) system and 24 V for ABS(12) and TPU(12) systems). In most cases, the maximum surface temperature significantly exceeded what can be considered a reasonable safety limit (55–60 °C) when considering the possibility that the final component can come into contact with the human skin. It is worth noting that the de-icing tests were carried out under hard conditions at a temperature of −25 °C with a thickness of 5 mm of consolidated ice. Milder environmental conditions closer to real winter ones would allow for shorter de-icing times. Furthermore, the temperature values reported for the heating film at constant DC voltage in Figure 11 were detected under the thermally insulating layer of the silicone vessel (see Figure 2).

Moreover, when planning activities of electronic control design, a discontinuous On–Off operation mode should be set in order for the smart components to work properly. This approach is to be preferred over using a lower applied voltage because at the maximum available electrical power the heating rate is as high as possible, meaning that the de-icing time is as low as possible.

It is worth noting that the TPV system was tested applying a lower voltage value of 12 V, as the same voltage applied for the other two systems (24 V) leads to very rapid and even excessive heating. In particular, it was detected that after only 2 min of an applied voltage of 24 V, a temperature value of 180 °C was reached.

## 4. Conclusions

Three thermoplastic matrices, namely, thermoplastic vulcanizate polymer (TPV), acrylonitrile butadiene styrene (ABS), and thermoplastic polyurethane (TPU), all of which are suitable for automotive applications, were mixed with CNT-based masterbatches through a single-screw (SS) extruder, obtaining nanocomposites with different loading levels of CNTs. Tensile and rheological measurements were performed, and it was found that the presence of CNTs strongly affects the tensile parameters and rheological properties of the formulated nanocomposites. The influence of the nanofiller depends on the amount of CNTs and the nature of the hosting matrix.

Electrothermal de-icing based on the Joule effect was performed on the developed samples.

A suitable electrical conductivity was obtained (σ ≈ 10^1^ S/m at 12%wt CNTs) for effective de-icing behaviour with low input voltages (12–24 V DC) safe for users. All materials resulted in useful de-icing times of less than 30′ to melt a 5 mm thick ice slab at −25 °C external temperature.

A simplified analytical model of the Joule effect arising from an energy balance of the system under study (heat equation) was developed. The analytical model fits well with the experimental data, in particular, the material final temperature, and can be used to predict the de-icing behaviour of materials of interest in any size/geometry and operating conditions, which is a very useful result for the purpose of preliminary component design.

## Figures and Tables

**Table 1:** Summary of pure thermoplastic polymers and CNT-based masterbatches adopted for developing electro-thermal plastic components.

Material	Neat Polymer Grade	CNT-Based Masterbatch Graphistrength^®^	Automotive Application
TPV (PP/EPDM)	Santoprene 121-58W175	C M14-25 (PP + 25% CNT)	Door gaskets
ABS	Starex SV-0167	C ABS1-17 (ABS + 17% CNT)	Windshield cowl, glass covers
TPU	Elastollan 1180 A 10	C TPU1-20 (TPU + 20% CNT)	Water circuit pipelines

**Table 2:** Fitting parameters used in analytical model of heating of sheet specimens.

Parameter	TPV 12% CNT	ABS 12% CNT	TPU 12% CNT
*T_ext_* [K]	298	298	298
*h_c_* [W/m^2^∙K]	10	10	10
*ρ* [kg/m^3^]	970 (TPV)	1040 (ABS)	1100 (TPU)
*V* [V]	12 or 24	12 or 24	12 or 24
*σ* [S/m]	89	51	23
*c_p_* [J/kg∙K]	1700 (polymers)	1700 (polymers)	1700 (polymers)
*L* [m]	0.089	0.089	0. 089
*b* [m]	0.044	0.044	0.044
*h* [m]	0.001416	0.000756	0.001116

## Data Availability

Data sharing not applicable.

## Supplementary Materials

The following supporting information can be downloaded at: https://www.mdpi.com/article/10.3390/nano12172979/s1, Figure S1: HAAKE RHEOMEX 302P single-screw extruder; Table S1: TPV nanocomposites produced by dilution of masterbatch C M14-25 (25% CNT); Table S2: ABS nanocomposites produced by dilution of masterbatch C ABS1-17 (17% CNT); Table S3: TPU nano-composites produced by dilution of masterbatch C TPU1-20 (20% CNT). Compounds extruded at 80 rpm are reported in italics. References [32,33] are cited in the supplementary materials.
