# Supplementary material for: Effective Practical Solutions for De-Icing of Automotive Component
Source: Nanomaterials (Basel). 2022 Aug 28;12(17):2979. doi: 10.3390/nano12172979 (PMC9457547; doi:10.3390/nano12172979)
Supplement: Supplementary file 1 [file nanomaterials-12-02979-s001.zip › nanomaterials-1806487-supplementary.pdf]

nanomaterials

MDPI

Supplementary Materials

Effective Practical Solutions for De-Icing of Automotive Component

Andrea Tinti [1,*], Gloria Anna Carallo [2], Antonio Greco [2], María Dolores Romero-Sánchez [3], Luigi Vertuccio [4] and Liberata Guadagno [5,*]

[1] Consorzio CETMA, Advanced Materials & Processes Consulting Department, , S.S. 7 km 706+030, 72100 Brindisi, Italy, andrea.tinti@cetma.it
[2] Department of Innovation Engineering, University of Salento, Via Monteroni, 73100 Lecce, Italy; gloria.carallo92@gmail.com (G.A.C.); antonio.greco@unisalento.it (A.G.)
[3] Applynano Solutions S.L., Parque Científico de Alicante, Naves de Apoyo 3, 03005 San Vicente, , Spain; md.romero@applynano.com
[4] Department of Engineering, University of Campania "Luigi Vanvitelli", Via Roma 29, 81031 Aversa, Italy; luigi.vertuccio@unicampania.it
[5] Department of Industrial Engineering, University of Salerno, Via Giovanni Paolo II, 84084 Fisciano, , Italy
* Correspondence: andrea.tinti@cetma.it (A.T.); lguadagno@unisa.it (L.G.)

Production of the nanocomposites and processing parameters

For each of the chosen matrices (TPV, ABS, and TPU), several compositions with varying CNT content have been produced by means of a thermoplastic melt process technology described below. All the analyzed compositions are shown in Tables S1, S2 and S3 for TPV, ABS and TPU respectively. Wmatrix is the weight percentage of the thermoplastic neat matrix, WCNT is the weight percentage of nanotubes into the final nano-composite, and Wmat,MB the weight percentage of the specific matrix of the masterbatch.

Nano-composites were produced by means of extrusion process, using a single-screw (SS) extruder HAAKE RHEOMEX 302P with a screw length/diameter (L/D) ratio of 30. The used die is characterized by a steel body, with a thin laminar slit in the front part (width = 10 cm), from which the melt exits. The thickness of the slit can be tuned by unscrewing threaded elements on the top of the die, as shown in Figure S1. In the present study, the settled thickness was 0.1 cm.

The processing parameters for the produced nano-composites, including the temperature profile from the feed zone to the die as well as the screw speed, are reported in Tables S1-S3.

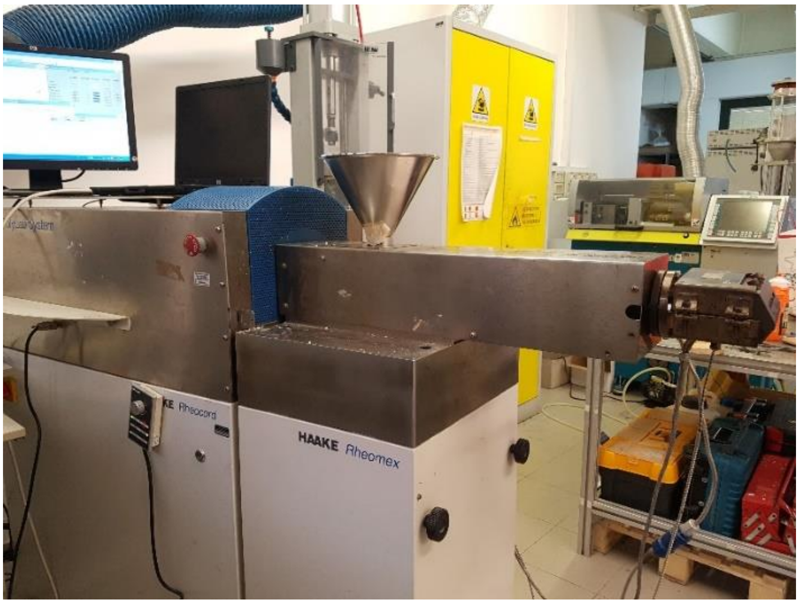

Figure S1. HAAKE RHEOMEX 302P single-screw extruder.

Table S1. TPV nano-composites produced by dilution of the masterbatch C M14-25 (25% CNT).

LABEL	W_{matrix}	W_{CNT}	$W_{mat,MB}$	Parameters
**TPV(0)**	100%	0%	0%	190 - 190 - 190 - 200 °C - 80 rpm
**TPV(3)**	88%	3%	9%	190 - 190 - 190 - 200 °C - 80 rpm
**TPV(6)**	76%	6%	18%	190 - 190 - 190 - 200 °C - 80 rpm
**TPV(9)**	64%	9%	27%	190 - 190 - 190 - 200 °C - 80 rpm
**TPV(12)**	52%	12%	36%	190 - 190 - 190 - 200 °C - 80 rpm
**C M14-25**	0%	25%	75%	190 - 190 - 190 - 200 °C - 80 rpm

Table S2. ABS nano-composites produced by dilution of the masterbatch C ABS1-17 (17% CNT).

LABEL	W_{matrix}	W_{CNT}	$W_{mat,MB}$	Parameters
**ABS(0)**	100%	0%	0%	225 - 225 - 230 - 240 °C - 30 rpm
**ABS(5)**	70.6%	5%	24.4%	225 - 225 - 230 - 240 °C - 30 rpm
**ABS(12)**	29.4%	12%	58.6%	225 - 225 - 230 - 240 °C - 30 rpm
**C ABS1-17**	0%	17%	83%	225 - 225 - 230 - 240 °C - 30 rpm

Table S3. TPU nano-composites produced by dilution of the masterbatch C TPU1-20 (20% CNT). In italics are reported compounds extruded at 80 rpm.

LABEL	W_{matrix}	W_{CNT}	$W_{mat,MB}$	Parameters
**TPU(0)**	100%	0%	0%	155 - 210 - 210 - 185 °C - 20 rpm
**TPU(0.5)**	97.5%	0.5%	2%	155 - 210 - 210 - 185 °C - 20 rpm
**TPU(1)**	95%	1%	4%	155 - 210 - 210 - 185 °C - 20 rpm
**TPU(1.5)**	93%	1.4%	5.6%	155 - 210 - 210 - 185 °C - 20 rpm
**TPU(2)**	91%	1.8%	7.2%	155 - 210 - 210 - 185 °C - 20 rpm
TPU(2)	*91%*	*1.8%*	*7.2%*	*155 - 210 - 210 - 185 °C - 80 rpm*
**TPU(2.2)**	89%	2.2%	8.8%	155 - 210 - 210 - 185 °C - 20 rpm
**TPU(2.5)**	87%	2.6%	10.4%	155 - 210 - 210 - 185 °C - 20 rpm
**TPU(4.5)**	77%	4.6%	18.4%	155 - 210 - 210 - 185 °C - 20 rpm
**TPU(6)**	69%	6.2%	24.8%	155 - 210 - 210 - 185 °C - 20 rpm
TPU(6)	*69%*	*6.2%*	*24.8%*	*155 - 210 - 210 - 185 °C - 80 rpm*
**TPU(7.5)**	62.5%	7.5%	30%	155 - 210 - 210 - 185 °C - 20 rpm
**TPU(12)**	40%	12%	48%	155 - 210 - 210 - 185 °C - 20 rpm
**C TPU1-20**	0%	20%	80%	155 - 210 - 210 - 185 °C - 20 rpm

Though twin screw extrusion was also tested as an efficient method to increase dispersion, the differences observed between single and twin extrusion processed films were not relevant. Mechanical and conductivity properties were not significantly improved by the use of a twin screw process. Therefore, single srew extrusion was used, since it allows for simultaneous compounding and forming.

Percolation theory

Different theoretical approaches have been proposed during the years on the study of percolation threshold values for nano-composites based on carbon nanotubes as nanofiller, proving great attention that this topic has gained all over scientific community. In general, percolation theory states that the percentage of CNTs affects electrical conductivity of nano-composites, i.e. increasing the content of nanotubes, conductivity should increase[1]. This evidence is proved by the formation of a conductive path, being nanotubes more and more in contact each other, that makes matrices – usually electrically insulating – conductive: the value that marks shifting in electrical properties is known as percolation threshold. It is correlated to a certain percentage of nanotubes and it also depends on pristine electrical behaviour of matrix. Then, statistical percolation model describes this relationship using the following formula:

$$\sigma_{nano} = \sigma_0 \left(\phi - \phi_{critical}\right)^t \quad \text{(S1)}$$

where σ_{nano} [S/m] is electrical conductivity of nano-composite, ϕ [%] is the weight fraction of nanotubes, $\phi_{critical}$ [%] is percolation threshold weight fraction, while σ_0 [S/m] and t [dimensionless] are proportionality coefficient and exponent of weight fraction percolation model, respectively [2]. For easiness of calculation, this formula can be converted in bi-logarithmic form:

$$log(\sigma_{nano}) = log(\sigma_0) + t \cdot log(\phi - \phi_{critical}) \quad \text{(S2)}$$

that can be parametrized as follows:

$$y = A + t \cdot x \quad \text{(S3)}$$

where A – i.e. $log(\sigma_0)$ – is intercept and t is angular coefficient of the linear dependence between electrical conductivity of nano-composite and difference of weight percentages of nanotubes, as stated by percolation threshold theory.

Theoretical percolation equation was used to fit experimental data in order to find all parameters of statistical percolation model, especially percolation thresholds.

References

1. Mansor, M.; Fadzullah, S.; Masripan, N.; Omar, G.; Akop, M. Comparison between functionalized graphene and carbon nanotubes: Effect of morphology and surface group on mechanical, electrical, and thermal properties of nanocomposites. In *Functionalized Graphene Nanocomposites and their Derivatives*; Elsevier, Amsterdam, Netherlands, 2019; pp. 177–204.
2. Battisti, A.; Skordos, A.A.; Partridge, I.K. Percolation threshold of carbon nanotubes filled unsaturated polyesters. *Compos. Sci. Technol.* **2010**, *70*, 633–637.
